# CircZNF124 regulates cell proliferation, leucine uptake, migration and invasion by miR‐199b‐5p/SLC7A5 pathway in endometrial cancer

**DOI:** 10.1002/iid3.477

**Published:** 2021-06-19

**Authors:** Liuping Shu, Yan Peng, Liyan Zhong, Xi Feng, Lifu Qiao, Yi Yi

**Affiliations:** ^1^ Department of Genecology, Wujin Hospital Affiliated with Jiangsu University The Wujin Clinical College of Xuzhou Medical University Changzhou China

**Keywords:** circZNF124, EC, miR‐199b‐5p, SLC7A5

## Abstract

**Background:**

Recent studies have revealed that circular RNA participates in endometrial carcinoma (EC) progression. Here we investigated the role of circRNA zinc finger protein 124 (circZNF124) in EC genesis and underlying mechanism.

**Methods:**

The expression levels of circZNF124, microRNA‐199b‐5p (miR‐199b‐5p) and solute carrier family 7 member 5 (SLC7A5) were detected by quantitative real‐time polymerase chain reaction. The expression of SLC7A5 and other indicated marker proteins was determined by western blot analysis. For functional assay, cell proliferation, leucine uptake and metastasis were investigated by total cell number, cell counting kit‐8, cell colony formation, leucine uptake or transwell assay. The interaction between miR‐199b‐5p and circZNF124 or SLC7A5 was predicted by starbase online database, and identified by mechanism assays. The impact of circZNF124 absence on tumor growth in vivo was revealed by xenograft mouse model assay. Immunohistochemistry assay was implemented to detect the positive expression rate of nuclear proliferation marker (Ki67).

**Results:**

CircZNF124 and SLC7A5 expression were significantly increased, while miR‐199b‐5p was decreased in EC tissues and cells compared with normal endometrial tissues or cells. CircZNF124 expression was closely associated with EC severity and lymph node metastasis. Additionally, circZNF124 depletion repressed cell proliferation, leucine uptake, migration and invasion in both HEC1A and Ishikawa cells. CircZNF124 regulated SLC7A5 expression by binding to miR‐199b‐5p. MiR‐199b‐5p inhibitors or SLC7A5 overexpression attenuated circZNF124 silencing‐mediated EC malignant progression. Furthermore, SLC7A5 absence inhibited tumor growth in vivo.

**Conclusion:**

CircZNF124 depletion inhibited EC cell malignancy by miR‐199b‐5p/SLC7A5 pathway, which demonstrated that circZNF124 had the potential as a therapeutic target for EC.

## Graphical abstract

CircZNF124 expression was related to severity of EC and lymph node metastasis. CircZNF124 silencing inhibited EC tumorigenesis by hindering leucine uptake through miR‐199b‐5p/SLC7A5 axis. This study demonstrates that circZNF124 can be employed as a diagnosis biomarker and circZNF124 inhibitors may be used as an anticancer drug for EC.

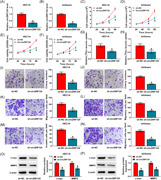

## 1 INTRODUCTION

Endometrial carcinoma (EC) ranks the fourth in incidence among gynecologic malignancies with an increasing trend in recent years.^1^ EC stage is commonly classified into stage I to stage IV via Federation International of Gynecology and Obstetrics (FIGO) based on the molecular genetic and clinicopathological characteristics.^2^ Stage I/II and stage III EC sufferers have more high survival rates with more than 70% and 55%, respectively, but survival rate of the stage IV EC patients is only 20%.^3^ At present, several molecular targeted treatments have not been used clinically owing to the unclear pathophysiological process of EC.^4^, ^5^ Thus, deeply exploring the genesis of EC will be urgent to develop new therapeutic targets for EC.

Circular RNA (circRNA) is an abundant conserved noncoding RNA produced by a back‐splicing process.^6^ An increasing number of research show that circRNA participates in regulating diverse biological behaviors of cells via sponging microRNA (miRNA), which possesses the binding sequence of RNA‐binding protein and modulates the transcription and translation of interest gene.^7^ In cancer progression, multiple circRNAs were involved. For example, circ_0000745 facilitated cervical cancer (CC) progression via regulating cell proliferation and metastasis,^8^ and promoted the development of acute lymphoblastic leukemia (ALL) via enhancing cell proliferative ability.^9^ Circ_0023404 played a promoting role in the progression of CC^10^ and lung carcinoma^11^ through accelerating cell proliferation and metastasis. Additionally, circRNA ATP binding cassette subfamily B member 10 (circABCB10) regulated the development of different cancers, such as breast cancer,^12^ lung cancer^13^ and thyroid cancer.^14^ However, there were few data about another circRNA, circRNA zinc finger protein 124 (circZNF124), in regulating cancer progression. According to the data from quantitative real‐time polymerase chain reaction (qRT‐PCR), we found circZNF124 was related to the severity of EC and lymph node metastasis. Moreover, cross‐sectional evidence has shown that circZNF124 is upregulated in extracellular vesicles from the serum of EC cases.^15^ However, whether circZNF124 is involved in EC progression remains unknown.

MiRNA is an evolutionarily conserved noncoding RNA with nearly 20 nucleotides, acting as an important regulator and taking part in cellular biological processes, such as cell proliferation, differentiation and amino acid transport.^16^, ^17^ MiRNA also plays vital parts in EC development. For example, miR‐522 enhanced cell proliferative, migratory and invasive capacities through targeting monoamine oxidase B.^18^ Shang et al.^19^ explained miR‐23b promoted cell proliferation, and repressed cell apoptosis and EC sensitivity to Cisplatin and Taxol. In addition, miRNA could control the expression of interest gene by mediating amino acid transport.^20^ Another miRNA, miR‐199b‐5p, has not been reported in EC genesis except inducing autophagic death.^21^ Based on bioinformatics predictions, we found miR‐199b‐5p was a potential target miRNA of circZNF124, and solute carrier family 7 member 5 (SLC7A5) was a possible target mRNA of miR‐199b‐5p. Thus, whether miR‐199b‐5p/SLC7A5 axis was responsible for the molecular mechanism underlying circZNF124 regulating EC progression was explored in the present study.

Herein, we detected circZNF124 expression in EC specimen tissues and cells, investigated the effects of circZNF124 silencing on EC progression in vitro and in vivo, and demonstrated whether circZNF124 mediated EC progression through miR‐199b‐5p/SLC7A5 axis.

## 2 MATERIALS AND METHODS

### 2.1 Tissue samples and the Ethics Committee

EC tissues (*N* = 46) and paracancerous healthy endometrial tissues (*N* = 46) were collected from EC sufferers underwent hysterectomy in Wujin Hospital Affiliated with Jiangsu University. The clinicopathologic features of endometrial cancer patients were listed in Table S1. All tissues were kept at −80°C. The histological diagnosis and FIGO stage of EC tissues were confirmed by two pathologists. The Ethics Committee of Wujin Hospital Affiliated with Jiangsu University approved this study. The subjects signed the written informed consent before surgery.

### 2.2 Cell culture

Human endometrial endothelial cell line (hEEC) and EC cell lines (HEC1A and Ishikawa) were purchased from EK‐Bioscience, and cultured in Dulbecco's modified Eagle's medium (DMEM; Procell) supplemented with 10% fetal bovine serum (FBS; Procell) as well as 1% penicillin/streptomycin (Procell) at 37°C in a humid incubator with 5% CO_2_.

### 2.3 Plasmid establishment, oligonucleotide synthesis and cell transfection

The small hairpin RNA against circZNF124 (sh‐circZNF124), the mimics of miR‐199b‐5p (miR‐199b‐5p), the inhibitors of miR‐199b‐5p (anti‐miR‐199b‐5p) and respective controls (sh‐NC, miR‐NC and anti‐NC) were provided by GenePharma. The overexpression plasmid of SLC7A5 was built by inserting the coding sequence of SLC7A5 into pcDNA 3.1(+) vector (pcDNA; EK‐Bioscience), and named as SLC7A5. Plasmids or oligonucleotides were incubated with cells after mixed with TurboFect reagent (Thermo Fisher Scientific). After 6 h, cell supernatant was removed and cells were cultured in DMEM containing 10% FBS (Procell) for the indicated time points. The oligonucleotide sequences were listed as below. Sh‐circZNF124 5′‐ATGAATAACTCGGTTGCCTTT‐3′, miR‐199b‐5p 5′‐CCCAGUGUUUAGACUAUCUGUUC‐3′, anti‐miR‐199b‐5p 5′‐GAACAGAUAGUCUAAACACUGGG‐3′, sh‐NC 5′‐CCTCTACCTGTCGCTGAGCTGTAAT‐3′, miR‐NC 5′‐UUUGUACUACACAAAAGUACUG‐3′ and anti‐NC 5′‐CAGUACUUUUGUGUAGUACAAA‐3′.

### 2.4 qRT‐PCR

Tissues and cells were lysed with TransZol (TransGen), and RNA was extracted with an RNAsimple kit (Tiangen). cDNA was synthesized with a FastKing RT Kit (Tiangen) or TQiagen reverse transcription kit (Hilden). To assess the expression of circZNF124, miR‐199b‐5p and SLC7A5, the obtained cDNA was mixed with FastFire qPCR PreMix (Tiangen), ROX Reference Dye (Tiangen) and primers (Tiangen), and then amplified with an ABI StepOne thermocycler (Thermo Fisher Scientific). The output values were analyzed with the 2‐ΔΔCt method. β‐Actin and U6 were employed for the normalization of circRNA/mRNA and miRNA expression, respectively. The sense and antisense primers were shown as following. CircZNF124 5′‐AGCCTTCAGTCGTTCCAG‐3′ and 5′‐TTCACAGCCACATCCTCA‐3′; miR‐199b‐5p 5′‐ACACTCCAGCTGGGCCCAGTGTTTAGACTAT‐3′ and 5′‐TGGTGTCGTGGAGTCG‐3′; SLC7A5 5′‐TAGGAGACAGAGCCAAGCAC‐3′ and 5′‐CACGGGAACAACAGAAACAA‐3′; β‐actin 5′‐CACCATTGGCAATGAGCGGTTC‐3′ and 5′‐AGGTCTTTGCGGATGTCCACGT‐3′; U6 5′‐CTCGCTTCGGCAGCACA‐3′ and 5′‐AACGCTTCACGAATTTGCGT‐3′.

### 2.5 Cell proliferation assays

To determine the proliferation of HEC1A and Ishikawa cells, total cell number assay, cell counting kit‐8 (CCK‐8) assay and cell colony formation assay were performed. In total cell number assay, HEC1A and Ishikawa cells were grown in 24‐well plates (5×10^4^ cells per well) and transfected with sh‐circZNF124, sh‐NC, miR‐199b‐5p, miR‐NC, anti‐miR‐199b‐5p, anti‐miR‐NC, SLC7A5 and pcDNA according to the defined purposes. The cells were counted every 24 h until the fourth day after transfection.

In CCK‐8 assay, cells were grown in 96‐well plates (3000 cells per well) for 16 h and transfected with plasmids or oligonucleotides. Cells were continued to be cultivated for 1, 2, 3 and 4 days, respectively. CCK‐8 solution (Beyotime) was added into each well and incubated for 4 h. Samples were assessed with a Synergy H1 Hyrbid microplate reader (BioTek) with wavelength at 450 nm.

For cell colony formation assay. Cells were cultured in six‐well plates (2×10^5^ cells per well) and transfected with plasmids or oligonucleotides. The cells were continued to be grown until the emergence of positive colonies. Then, cell supernatant was discarded, and methanol (Beyotime) and crystal violet (Beyotime) were incubated with the cells. Finally, cell colony‐forming ability was determined by assessing the number of colonies. A colony was deemed when cell numbers over 50.

### 2.6 Leucine uptake assay

To determine the leucine uptake in EC cells, [^3^H]‐labelled leucine uptake assay was performed as shown previously.^22^ In brief, HEC1A and Ishikawa cells were grown in 96‐well plates with leucine‐free DMEM (MP Biomedicals) and [^3^H]‐l‐leucine (PerkinElmer) for 15 min. After that, the cultured cells were harvested and transferred to filter paper. Then, scintillation fluid (PerkinElmer) was incubated with the cells, and the samples were assessed using a liquid scintillation counter (PerkinElmer).

### 2.7 Transwell assay

Cell migration and invasion were investigated by transwell chambers (Millipore). Specially, the chambers were pre‐incubated with Matrigel (Corning) to reveal cell invasive ability. Briefly, cells were homogenized in serum‐free DMEM (Procell) and placed into the upper chambers. DMEM containing 15% FBS (Procell) was added into the lower chambers. After 24 h, the cells were incubated with methanol (Beyotime) and crystal violet (Beyotime), respectively. Results were confirmed by figuring up the number of cells in the lower chambers with an Eclipse Ts2R inverted microscope (Nikon) at a × 100 magnification.

### 2.8 Target prediction and dual‐luciferase reporter assay

The binding sites between miR‐199b‐5p and circZNF124 or SLC7A5 were predicted by starbase online database (http://starbase.sysu.edu.cn/agoClipRNA.php?source=circRNA%26flag=none%26clade=mammal%26genome=human%26assembly=hg19%26miRNA=all%26clipNum=1%26deNum=0%26target=hsa_circ_0005667 or http://starbase.sysu.edu.cn/agoClipRNA.php?source=mRNA%26flag=miRNA%26clade=mammal%26genome=human%26assembly=hg19%26miRNA=hsa-miR-199b-5p%26clipNum=%26deNum=%26panNum=%26proNum=%26program=%26target=), respectively. The wild‐type (WT) plasmids of circZNF124 (WT‐circZNF124) and the 3′‐untranslated region (3′‐UTR) of SLC7A5 (WT‐SLC7A5 3′‐UTR), and the respective mutant (MUT) plasmids (MUT1‐circZNF124, MUT2‐circZNF124, MUT1&2‐circZNF124, MUT‐SLC7A5 3′‐UTR) were provided by Geneseed Co., Ltd. Then, the plasmids premixed with both TurboFect reagent (Thermo Fisher Scientific) and miR‐199b‐5p or miR‐NC were added into the culture wells, and incubated with cells based on the instruction of manufacture. Finally, *firefly* luciferase activity was tested with a Dual‐Lucy Assay Kit with *Renilla* luciferase activity as a control. WT‐SLC7A5 3′‐UTR contained WT‐SLC7A5 3′‐UTR site1, WT‐SLC7A5 3′‐UTR site2 and WT‐SLC7A5 3′‐UTR site3. MUT‐SLC7A5 3′‐UTR contained MUT‐SLC7A5 3′‐UTR site1, MUT‐SLC7A5 3′‐UTR site2 and MUTT‐SLC7A5 3′‐UTR site3.

### 2.9 RNA immunoprecipitation (RIP) assay

The interaction between miR‐199b‐5p and circZNF124 or SLC7A5 was confirmed using a Magna RIP kit (Millipore). Briefly, the cultured HEC1A and Ishikawa cells were lysed with RIP lysis buffer (Millipore). The lysates were incubated with the magnetic beads coated with anti‐argonaute2 (anti‐Ago2; Abcam, Cambridge, UK) and anti‐immunoglobulin G (anti‐IgG; Abcam), respectively. After the beads were washed, both circZNF124 and miR‐199b‐5p enrichment in co‐precipitated RNAs were detected by qRT‐PCR.

### 2.10 RNA pull‐down assay

The association between circZNF124 and miR‐199b‐5p was further identified by RNA pull‐down assay. In brief, biotin‐labeled miR‐199b‐5p (Biotin‐miR‐199b‐5p; GenePharma) and miR‐NC (Biotin‐NC; GenePharma) were transfected into both HEC1A and Ishikawa cells, and the cells were cultured for another 48 h. Then, the cells were lysed and the lysates were incubated with streptavidin‐coupled beads (Invitrogen) for 4 h. The complex pulled down by the beads was detected by qRT‐PCR.

### 2.11 Western blot analysis

Cells and tissues were lysed with NP‐40 buffer (Beyotime), and the lysates mixed with loading buffer (Thermo Fisher Scientific) were denatured at 95°C. The mixtures were loaded onto 12% bis‐tris‐acrylamide gels (Thermo Fisher Scientific), and wet‐transferred onto polyvinylidene fluoride membranes (Millipore). After blocking with defatted dry milk (Solarbio), the membranes were incubated with anti‐c‐myc (1:2000; Affinity), anti‐matrix metalloprotein 2 (anti‐MMP2) (1:1000; Affinity), anti‐SLC7A5 (1:2000; Affinity) and anti‐β‐actin (1:8000; Affinity), followed by the incubation with secondary antibody (1:10,000; Affinity). The protein bands were visualized with RapidStep ECL Reagent (Millipore), and protein expression was quantified by ImageJ software (NIH) with β‐actin as a reference.

### 2.12 Xenograft mouse model assay

Female BALB/c nude mice (6 weeks of age) were purchased from Charles River, and fed in pathogen‐free environment. All mice were divided into 2 groups (sh‐circZNF124 group and sh‐NC group, *N* = 5 per group). 2×10^6^ Ishikawa cells stably transfected with sh‐circZNF124 or sh‐NC were hypodermically injected into the right side of mice back. After 7 days, tumor volume was measured every 1 week until the 35th day after injection. All mice were euthanatized, and the forming tumors were excised and weighed. In addition, a part of each tumor was kept for the expression analysis of circZNF124, miR‐199b‐5p and SLC7A5. This study was permitted by the Animal Care and Use Committee of Wujin Hospital Affiliated with Jiangsu University. The volume was calculated according to the formula: volume (mm^3^) = width^2^ × length/2.

### 2.13 Immunohistochemistry (IHC) assay

The nuclear proliferation marker (Ki67)‐positive cells in the forming tissues from xenograft mouse model assay were detected by IHC assay. In brief, the tissues were cut into the sections, and embedded into paraffin. After performing heating at 60°C for 20 min, the sections were deparaffinized and hydrated with xylene (Millipore) and ethanol (Millipore), respectively. Antigen retrieval was conducted by heating the sections immersed in the sodium citrate (Millipore). Following that, Hydrogen Peroxide (Millipore) was employed to incubate with the sections. The sections were incubated with anti‐Ki67 (1:100; Affinity) and goat anti‐rabbit IgG (1:200; Affinity), respectively. 3,3‐diaminobenzidine (Abcam) and hematoxylin (Millipore) were incubated with the tissues, and the staining images were captured with a microscope (Olympus).

### 2.14 Statistical analysis

All data were obtained based on three independent duplicate tests and analyzed with SPSS software (IBM). Results were shown as means ± standard deviations (*SD*). The significant differences were analyzed with two‐tailed Student's *t*‐tests or Wilcoxon rank‐sum test between the two groups, and were assessed with one‐way analysis of variance (ANOVA) between more than two groups. *p* value < .05 was considered as statistical significance.

## 3 RESULTS

### 3.1 CircZNF124 expression was upregulated in EC tissues and cells, and associated with FIGO stage and lymph node metastasis

**Figure 1:**
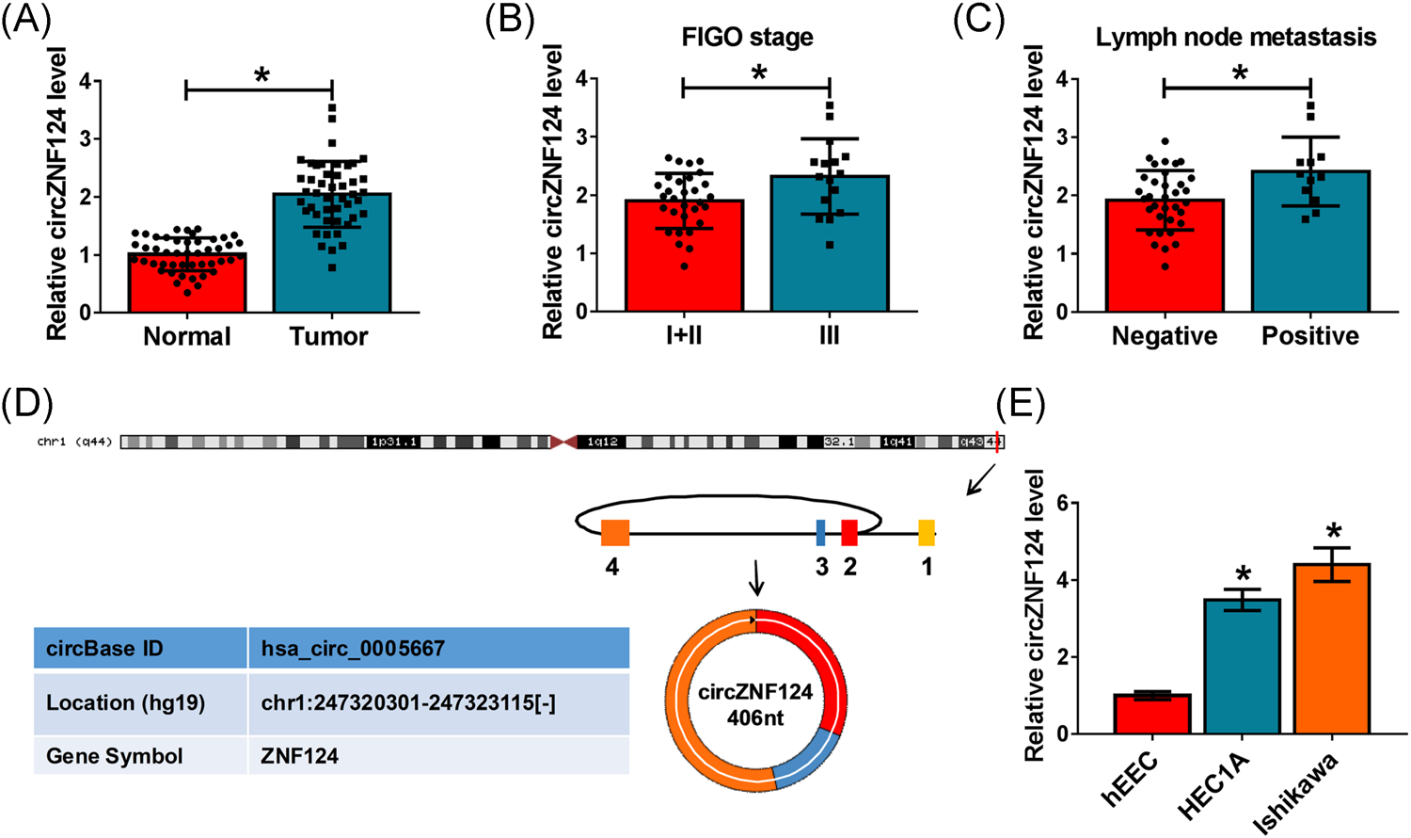
CircZNF124 expression in EC tissues and cells and its correlation with FIGO stage and lymph node metastasis. (A) CircZNF124 expression was determined by qRT‐PCR in 46 pairs of EC tissues and paracancerous normal endometrial tissues. (B) CircZNF124 expression was detected by qRT‐PCR in stage I + II EC tissues (*N* = 30) and stage III EC tissues (*N* = 16). (C) CircZNF124 expression was detected by qRT‐PCR in positive lymph node metastasis tissues (*N* = 12) and negative lymph node metastasis tissues (*N* = 34). (D) Schematic illustration demonstrated circZNF124 formation via exons 2–4 circularization of the ZNF124 gene, and that circZNF124 was located in chrl:247320301‐247323115[‐]. (E) CircZNF124 expression was determined by qRT‐PCR in HEC1A, Ishikawa and hEEC cells. Chrl, chromosome 1. The comparisons were assessed with Wilcoxon rank‐sum test in (A–C) and with ANOVA in (E). *p < .05. ANOVA, analysis of variance; Chrl, chromosome 1; circZNF124, circRNA zinc finger protein 124; EC, endometrial carcinoma; FIGO, Federation International of Gynecology and Obstetrics; hEEC, Human endometrial endothelial cell line; qRT‐PCR, quantitative real‐time polymerase chain reaction

The expression property of circZNF124 was firstly determined. Results showed circZNF124 expression was higher in EC tissues than in normal endometrial tissues (Figure 1A). Meanwhile, qRT‐PCR results presented that circZNF124 expression was increased in stage III EC tissues in comparison with its expression in stage I + II EC tissues (*p* < .05) (Figure 1B), and also upregulated in positive lymph node metastasis tissues when compared with negative lymph node metastasis tissues (*p* < .05) (Figure 1C). Additionally, we found circZNF124 was located in chrl:247320301‐247323115[‐] and was composed of exons 2–4 of ZNF124 gene (Figure 1D). Furthermore, we found circZNF124 expression was increased in EC cell lines (HEC1A and Ishikawa) as compared with human endometrial endothelial cells (hEEC) (Figure 1E). These data demonstarted that circZNF124 might be involved in EC progression.

### 3.2 CircZNF124 silencing repressed cell proliferation, leucine uptake, migration and invasion in HEC1A and Ishikawa cells

**Figure 2:**
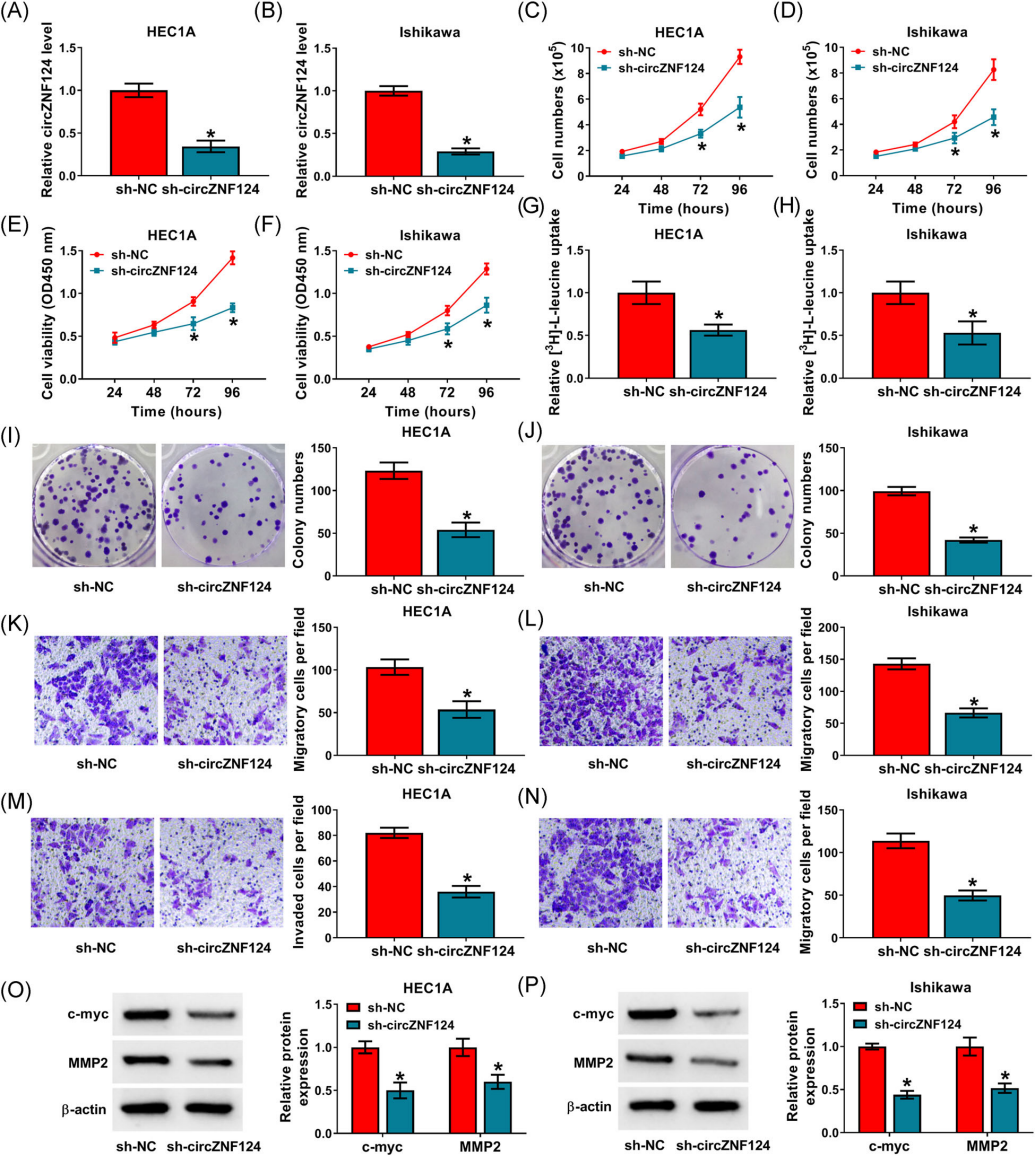
CircZNF124 regulated EC cell malignancy and leucine uptake. (A–P) HEC1A and Ishikawa cells were transfected with sh‐circZNF124 and sh‐NC, respectively. (A and B) CircZNF124 expression was detected by qRT‐PCR. (C and D) Cell numbers were determined by total cell number assay. (E and F) Cell viability was detected by CCK‐8 assay. (G and H) Leucine uptake assay was performed to detect the consumption of leucine. (I and J) Cell colony formation assay was carried out to detect cell colony‐forming ability. (K–N) The migration and invasion of both HEC1A and Ishikawa cells were investigated by transwell assay. (O and P) The protein expression of c‐myc and MMP2 was detected by western blot analysis. The comparisons were assessed with two‐tailed Student's *t* tests. *p < .05. CCK‐8, cell counting kit‐8; circZNF124, circRNA zinc finger protein 124; EC, endometrial carcinoma; MMP2, matrix metalloproteinase 2; qRT‐PCR, quantitative real‐time polymerase chain reaction

The role of circZNF124 in EC progression was continued to be explored in vitro. Results firstly showed circZNF124 expression was significantly reduced in both the HEC1A and Ishikawa cells transfected with sh‐circZNF124 as compared with these cells transfected with sh‐NC (Figure 2A,B), which suggested that sh‐circZNF124 was effective in downregulating circZNF124 expression. Additionally, the number of HEC1A and Ishikawa cells was significantly decreased after circZNF124 knockdown (Figure 2C,D). Cell viability and cell colony‐forming ability were inhibited after circZNF124 silencing in both HEC1A and Ishikawa cells (Figure 2E,F,I,J). These results demonstrated circZNF124 knockdown repressed the proliferation of HEC1A and Ishikawa cells. Additionally, circZNF124 downregulation hindered leucine uptake in HEC1A and Ishikawa cells (Figure 2G,H). Transwell assay exhibited that circZNF124 absence inhibited cell migration and invasion (Figure 2K–N). Furthermore, circZNF124 depletion reduced the expression of proliferation‐related protein (c‐myc) and metastasis‐related protein (MMP2) (Figure 2O,P), which suggested the repressive roles of circZNF124 knockdown in the proliferation and metastasis of EC cells. Thus, the above data explained that circZNF124 knockdown restrained EC cell proliferation, migration, invasion and leucine uptake.

### 3.3 CircZNF124 acted as a sponge of miR‐199b‐5p in EC cells

**Figure 3:**
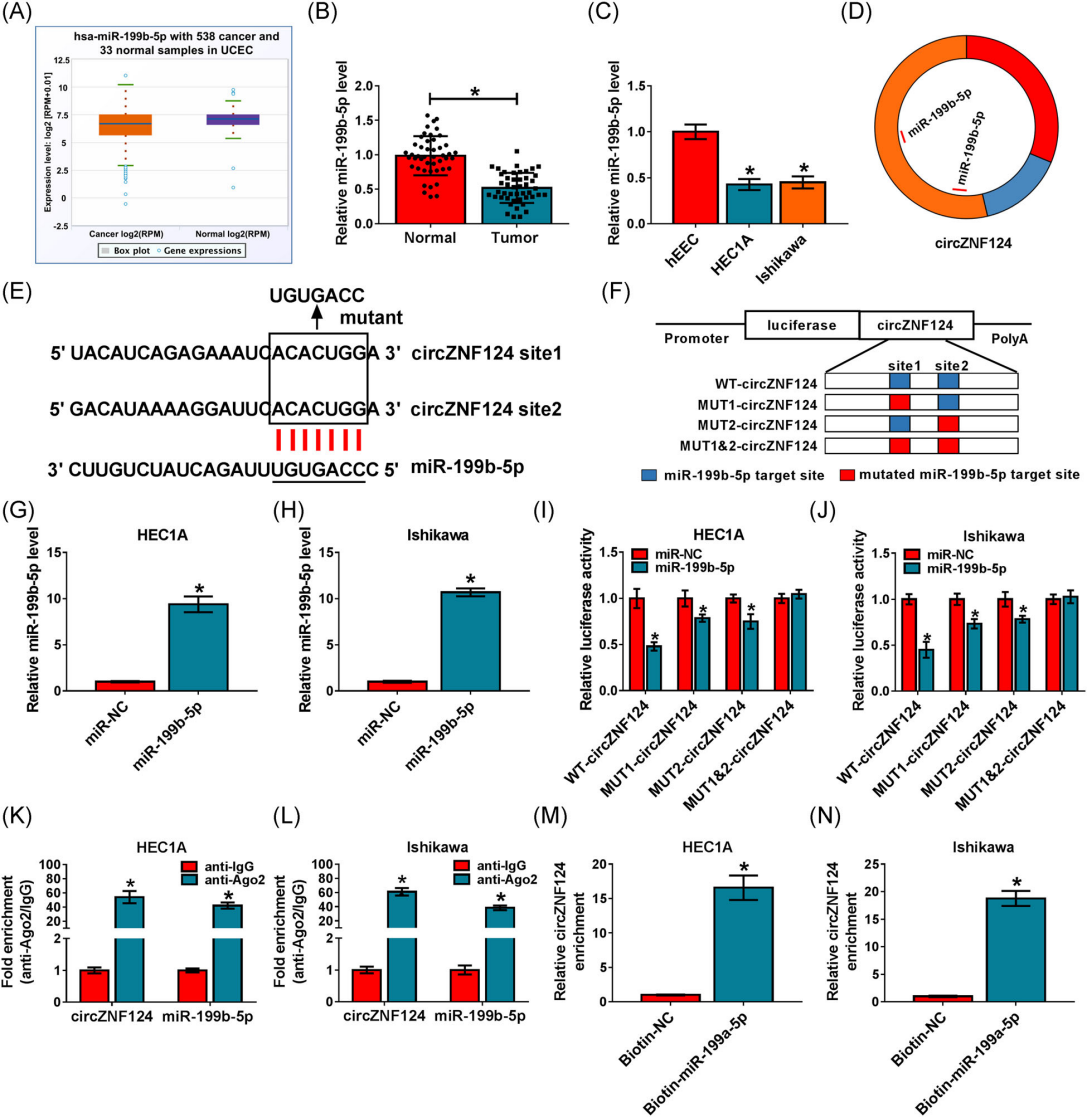
CircZNF124 was directly associated with miR‐199b‐5p. (A) TCGA data set was performed to assess miR‐199b‐5p expression in UCEC tissues (*N* = 538) and normal endometrial tissues (*N* = 33). (B and C) MiR‐199b‐5p expression was determined by qRT‐PCR in EC tissues (*N* = 46), paracancerous normal endometrial tissues (*N* = 46) as well as hEEC, HEC1A and Ishikawa cells. (D) Schematic illustration showed the exon 4 of circZNF124 contained two binding sequences of miR‐199b‐5p. (E) The binding sites between circZNF124 and miR‐199b‐5p were predicted by starbase online database. (F) Schematic illustration presented the mutant sites of circZNF124 sequence. (G and H) The overexpression efficiency of miR‐199b‐5p was determined by qRT‐PCR in HEC1A and Ishikawa cells. (I–N) Dual‐luciferase reporter, RIP and RNA pull‐down assays were conducted to explain that circZNF124 was directly associated with miR‐199b‐5p. RPM, reads per million mapped reads. The significant differences were compared with Wilcoxon rank‐sum test in (B), with ANOVA in (c), and with two‐tailed Student's *t* tests in (G–N). *p < .05. ANOVA, analysis of variance; circZNF124, circRNA zinc finger protein 124; EC, endometrial carcinoma; hEEC, human endometrial endothelial cell line; qRT‐PCR, quantitative real‐time polymerase chain reaction; RIP, RNA immunoprecipitation; TCGA, The Cancer Genome Atlas; UCEC, uterine corpus endometrial carcinoma

The study continued to demonstrate the mechanism by which circZNF124 regulated EC progression. Given that circRNA contained miRNA response elements (MREs), the miRNAs associated with circZNF124 were further screened. The data from qRT‐PCR showed miR‐199b‐5p, a candidate, was lowly expressed in uterine corpus endometrial carcinoma (UCEC) tissues compared with normal endometrial tissues (Figure 3A). Meanwhile, qRT‐PCR data showed miR‐199b‐5p was weakly expressed in EC tissues, HEC1A cells and Ishikawa cells as compared to normal endometrial tissues and hEEC cells, respectively (Figure 3B,C). Also, the study exhibited the low miR‐199b‐5p expression in stage III EC tissues in comparison with stage I + II EC tissues (Figure S1a). We then found that there were 2 complementary sequences between circZNF124 and miR‐199b‐5p (Figure 3D). The above data implied that miR‐199b‐5p might be associated with circZNF124. To prove the interaction, the sites of circZNF124 bound by miR‐199b‐5p were mutated and the mutated sites were shown in Figure 3E,F. Results presented that miR‐199b‐5p was effective in upregulating miR‐199b‐5p expression (Figure 3G,H). Subsequently, dual‐luciferase reporter assay showed miR‐199b‐5p mimics repressed the luciferase activity of WT‐circZNF124, MUT1‐circZNF124 and MUT2‐circZNF124, but not the luciferase activity of MUT1&2‐circZNF124 (Figure 3I,J). RIP assay presented both circZNF124 and miR‐199b‐5p were significantly enriched by anti‐Ago2 compared with them in anti‐IgG group (Figure 3K,L). RNA pull‐down assay also displayed that circZNF124 expression was higher in Biotin‐miR‐199b‐5p group than in Biotin‐NC group (Figure 3M,N). The above evidence demonstrated circZNF124 was directly associated with miR‐199b‐5p.

### 3.4 MiR‐199b‐5p mimics inhibited cell proliferation, leucine uptake, migration and invasion in EC cells

**Figure 4:**
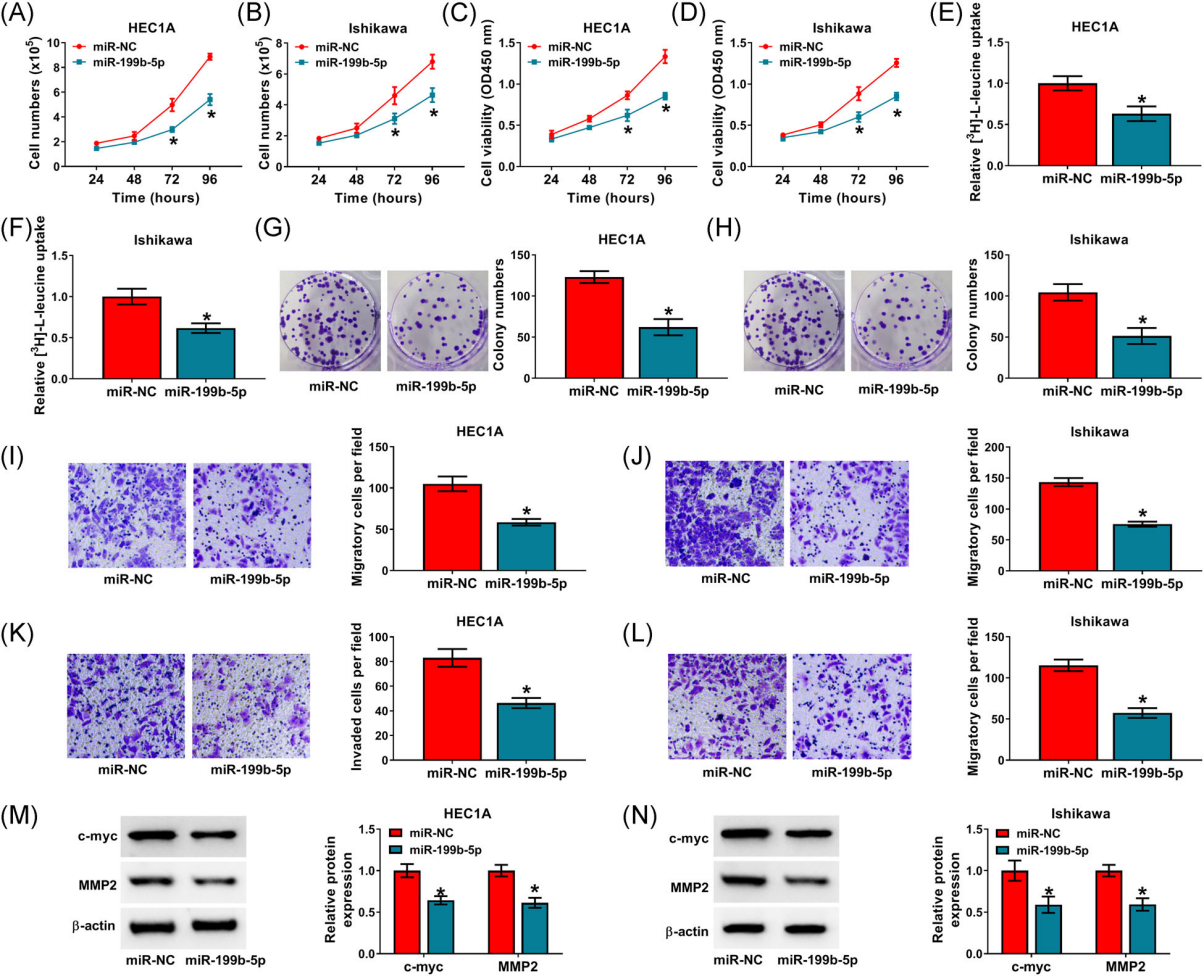
MiR‐199b‐5p regulated the proliferation, leucine uptake, migration and invasion of EC cells. (A–N) HEC1A and Ishikawa cells were transfected with miR‐199b‐5p or miR‐NC. (A and B) Cell numbers of HEC1A and Ishikawa cells were determined by total cell number assay. (C and D) Cell viability was detected through CCK‐8 assay. (E and F) Leucine uptake assay was employed to reveal the consumption of leucine. (G and H) Cell colony‐forming ability was determined by cell colony formation assay. (I–L) Cell migratory and invasive abilities were investigated by transwell assay. (M and N) Western blot analysis was employed to detect the protein expression of c‐myc and MMP2. The comparisons were assessed with two‐tailed Student's *t* tests. *p < .05. CCK‐8, cell counting kit‐8; EC, endometrial carcinoma; MMP2, matrix metalloproteinase 2

The effects of miR‐199b‐5p on the biological behaviors of EC cells were further revealed. Results showed miR‐199b‐5p mimics significantly decreased the cell numbers and cell viability of HEC1A and Ishikawa cells (Figure 4A–D). The leucine uptake also was repressed after upregulation of miR‐199b‐5p (Figure 4E,F). Additionally, cell colony‐forming ability was repressed by increased expression of miR‐199b‐5p (Figure 4G,H). Furthermore, miR‐199b‐5p mimics restrained the migration and invasion of both HEC1A and Ishikawa cells (Figure 4I–L). The protein expression of c‐myc and MMP2 was also downregulated by miR‐199b‐5p mimics (Figure 4M,N). All results suggested miR‐199b‐5p inhibited EC cell malignancy in vitro.

### 3.5 CircZNF124 silencing decreased SLC7A5 expression by interacting with miR‐199b‐5p in EC cells

**Figure 5:**
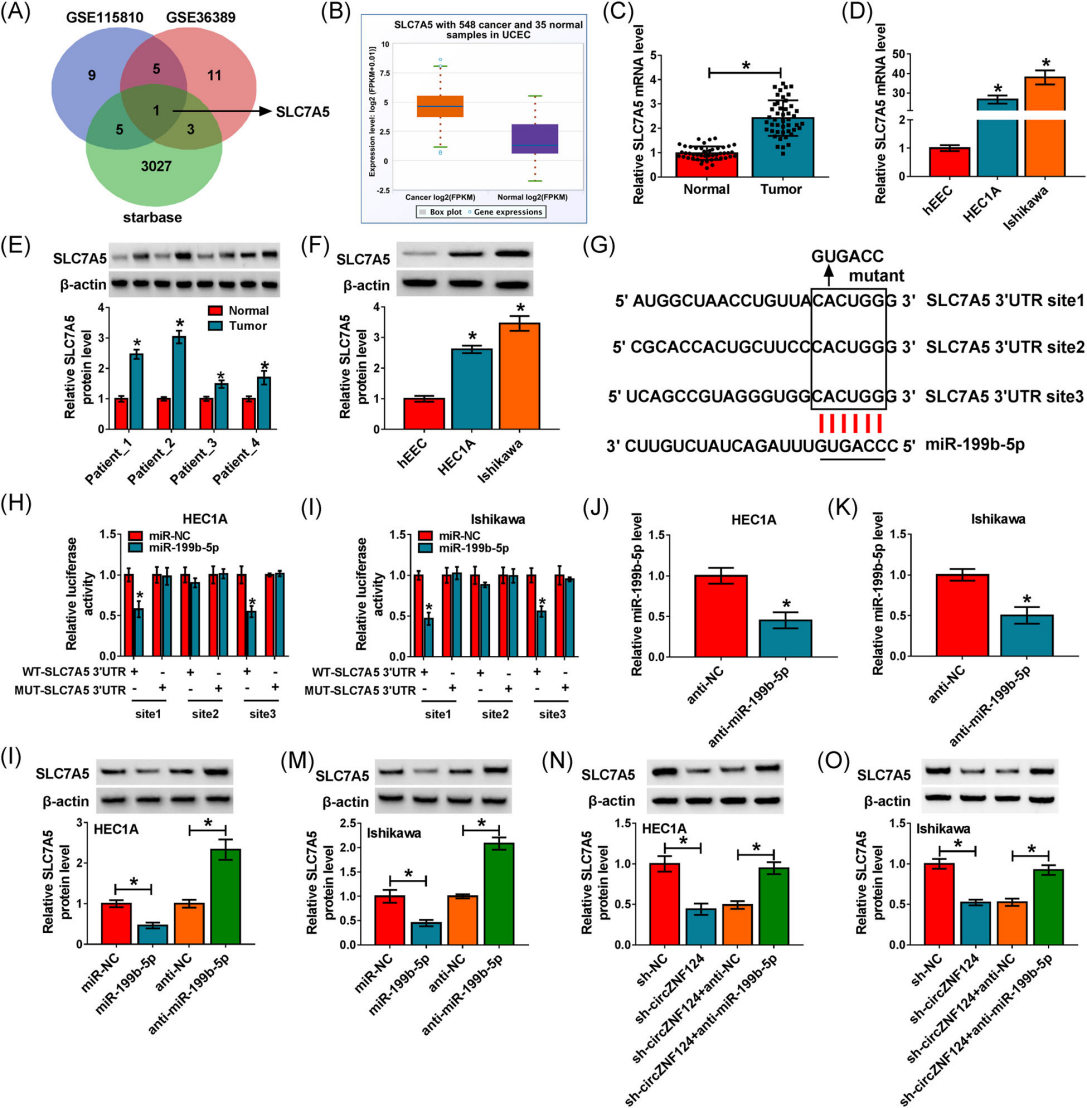
CircZNF124 modulated SLC7A5 expression by binding to miR‐199b‐5p in EC cells. (A) The mRNAs upregulated in both GSE115810 and GSE36389 datasets and bound by miR‐199b‐5p were assessed by Venn Diagram analysis. (B) TCGA data set was employed to predict SLC7A5 expression in UCEC tissues (*N* = 548) and normal endometrial tissues (*N* = 35). (C–F) The mRNA and protein expression of SLC7A5 were detected by qRT‐PCR and western blot analysis, respectively, in EC tissues (*N* = 46), paracancerous normal endometrial tissues (*N* = 46) as well as hEEC, HEC1A and Ishikawa cells. (G) The binding sites between miR‐199b‐5p and SLC7A5, and mutant sites of SLC7A5 3′‐UTR site1, SLC7A5 3′‐UTR site2 and SLC7A5 3′‐UTR site3 were presented. (H and I) The putative targeting relationship between miR‐199b‐5p and SLC7A5 was proved by dual‐luciferase reporter assay in HEC1A and Ishikawa cells. (J and K) MiR‐199b‐5p expression was detected by qRT‐PCR in both the HEC1A and Ishikawa cells transfected with anti‐miR‐199b‐5p or anti‐NC. (L and M) Western blot analysis was employed to determine the effects of miR‐199b‐5p mimics and inhibitors on SLC7A5 protein expression in HEC1A and Ishikawa cells. (N and O) The impacts between circZNF124 silencing and miR‐199b‐5p inhibitors on SLC7A5 protein expression were revealed by western blot analysis. The significant differences were compared with Wilcoxon rank‐sum test in (C), with ANOVA in (D, F, L, M N, O), and with two‐tailed Student's *t* tests in (E, H, I, J and K). *p < .05. 3′‐UTR, 3′‐untranslated region; ANOVA, analysis of variance; circZNF124, circRNA zinc finger protein 124; EC, endometrial carcinoma; hEEC, human endometrial endothelial cell line; mRNA, messenger RNA; qRT‐PCR, quantitative real‐time polymerase chain reaction; SLC7A5, solute carrier family 7 member 5; TCGA, The Cancer Genome Atlas; UCEC, uterine corpus endometrial carcinoma

To seek the mRNA bound to miR‐199b‐5p, GSE115810 (https://www.ncbi.nlm.nih.gov/geo/query/acc.cgi?acc=GSE115810) and GSE36389 datasets (https://www.ncbi.nlm.nih.gov/geo/query/acc.cgi?acc=GSE36389) as well as starbase online database were employed. Results showed that there were only 9 mRNAs that were upregulated in GSE115810 or GSE36389 datasets and contained the binding sites of miR‐199b‐5p. Venn Diagram analysis further presented that only SLC7A5 was upregulated in both the GSE115810 and GSE36389 datasets and possessed the putative binding sequences of miR‐199b‐5p among the 9 mRNAs (Figure 5A). It was subsequently found that SLC7A5 was upregulated in UCEC tissues compared with normal endometrial tissues through The Cancer Genome Atlas (TCGA) data set (Figure 5B), but had no apparent change in stage III EC tissues when compared with stage I + II EC tissues (Figure S1b). Meanwhile, data presented SLC7A5 was overexpressed in EC tissues as well as EC cells (HEC1A and Ishikawa) when compared with normal endometrial tissues and hEEC cells, respectively (Figure 5C–F). The above data suggested that SLC7A5 might be targeted by miR‐199b‐5p. To identify the association between miR‐199b‐5p and SLC7A5, the putative binding sites of SLC7A5 for miR‐199b‐5p were mutated, and the mutated sites were shown in Figure 5G. Subsequently, we found the luciferase activity of WT‐SLC7A5 3′‐UTR site1 and WT‐SLC7A5 3′‐UTR site3 was substantially repressed, but the luciferase activity of WT‐SLC7A5 3′‐UTR site2 had little change when compared with the luciferase activity of mutant SLC7A5 3′‐UTR (Figure 5H,I) after overexpression of miR‐199b‐5p, suggesting that miR‐199b‐5p interacted with SLC7A5 by binding to SLC7A5 3′‐UTR site1 or SLC7A5 3′‐UTR site3. Additionally, qRT‐PCR analysis showed the high efficiency of anti‐miR‐199b‐5p in reducing miR‐199b‐5p expression (Figure 5J,K). The data from Figure 5L,M showed miR‐199b‐5p mimics decreased SLC7A5 protein expression, and miR‐199B‐5p inhibitors increased SLC7A5 protein expression. Furthermore, we found that circZNF124 silencing reduced SLC7A5 protein expression, whereas the reduced expression of SLC7A5 was reversed after the combination of circZNF124 knockdown and miR‐199b‐5p downregulation (Figure 5N,O). Thus, all evidence demonstrated circZNF124 regulated SLC7A5 expression by binding to miR‐199b‐5p.

### 3.6 CircZNF124 silencing hindered cell proliferation, leucine uptake, migration and invasion by regulating miR‐199b‐5p and SLC7A5 in EC cells

**Figure 6:**
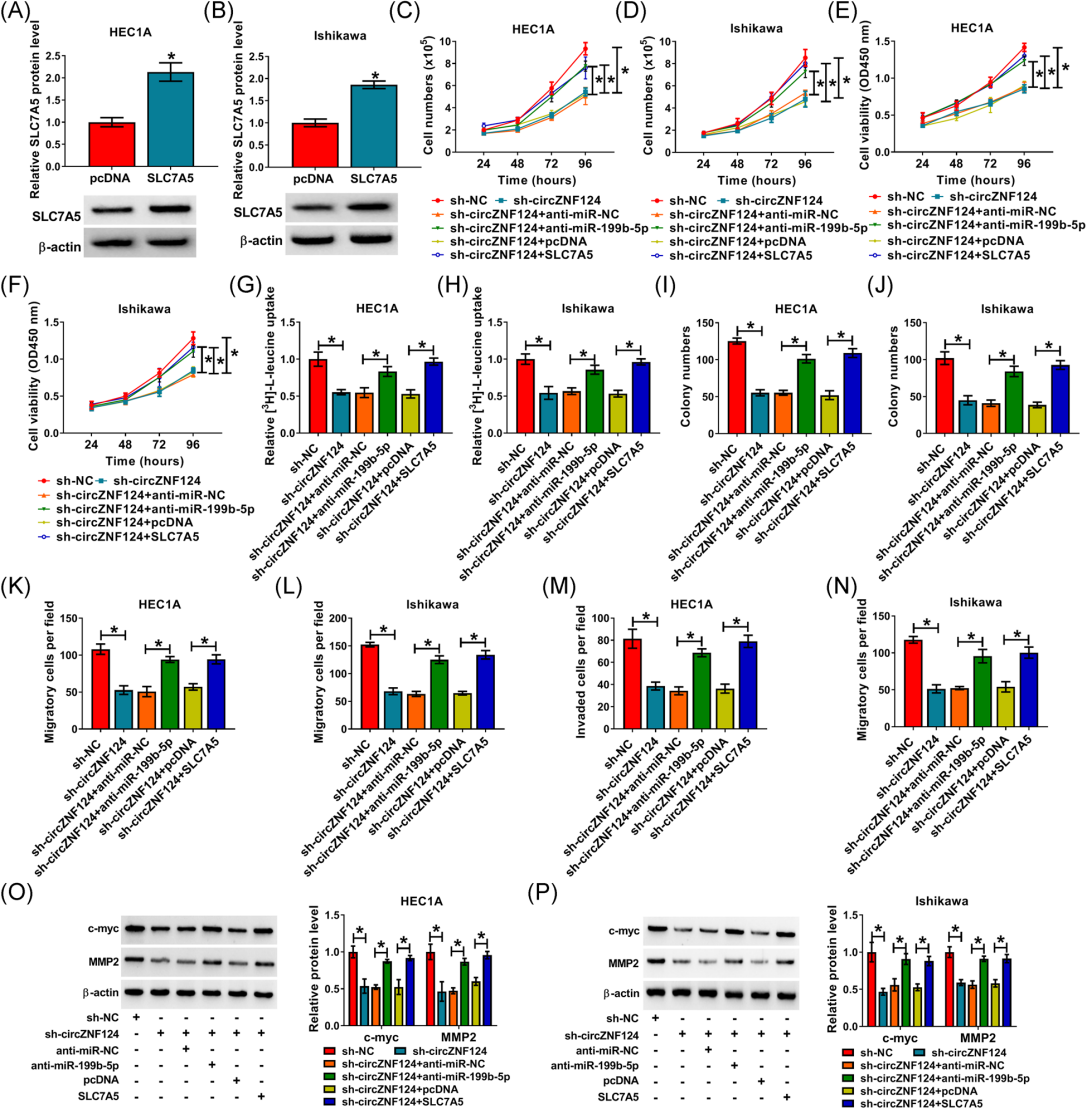
CircZNF124 absence repressed cell proliferation, leucine uptake, migration and invasion by regulating miR‐199b‐5p and SLC7A5. (A and B) SLC7A5 protein expression was detected by western blot analysis in both the HEC1A and Ishikawa cells transfected with pcDNA or SLC7A5. (C–P) HEC1A and Ishikawa cells were transfected with sh‐NC, sh‐circZNF124, sh‐circZNF124+anti‐miR‐NC, sh‐circZNF124+anti‐miR‐199b‐5p, sh‐circZNF124+pcDNA and sh‐circZNF124 + SLC7A5, respectively. (C and D) Cell numbers were determined by total cell number assay. (E and F) Cell viability was detected by CCK‐8 assay. (G and H) Leucine uptake assay was performed to determine leucine uptake. (I and J) Cell colony formation assay was carried out to detect cell colony‐forming ability. (K–N) Transwell assay was employed to detect the migration and invasion of HEC1A and Ishikawa cells. (O and P) The protein expression of c‐myc and MMP2 was detected by western blot analysis. The significant differences were compared with two‐tailed Student's *t*‐tests in (A and B), and with ANOVA in (C–P). *p < .05. ANOVA, analysis of variance; CCK‐8, cell counting kit‐8; circZNF124, circRNA zinc finger protein 124; MMP2, matrix metalloproteinase 2; SLC7A5, solute carrier family 7 member 5

Given the relationship between circZNF124 and miR‐199b‐5p or SLC7A5, whether circZNF124 silencing repressed biological behaviors of EC cells by modulating miR‐199b‐5p and SLC7A5 was revealed in this part. Results initially presented that SLC7A5 was effective in upregulating SLC7A5 expression (Figure 6A,B). Subsequently, the repressive impacts of circZNF124 silencing on cell numbers and cell viability were reversed by miR‐199b‐5p inhibitors or SLC7A5 overexpression (Figure 6C–F). The inhibitory influence of circZNF124 silencing on leucine uptake was also attenuated by reduced miR‐199b‐5p or upregulated SLC7A5 (Figure 6G,H). Additionally, colony formation assay presented that the inhibitory impact of circZNF124 silencing on cell colony‐forming ability was restrained by miR‐199b‐5p inhibitors or ectopic SLC7A5 expression (Figure 6I,J). Transwell assay also showed circZNF124 knockdown suppressed cell migration and invasion, but the reduced migration and invasion by circZNF124 absence were relieved after the combined action of circZNF124 silencing and miR‐199b‐5p inhibitors or SLC7A5 overexpression (Figure 6K–N). The decreased protein expression of c‐myc and MMP2 caused by circZNF124 knockdown were also attenuated by miR‐199b‐5p inhibitors or SLC7A5 overexpression (Figure 6O,P). All in all, all evidence demonstrated circZNF124 regulated EC cell malignancy by controlling miR‐199b‐5p and SLC7A5 expression.

### 3.7 CircZNF124 absence suppressed tumor formation by regulating miR‐199b‐5p and SLC7A5 in vivo

**Figure 7:**
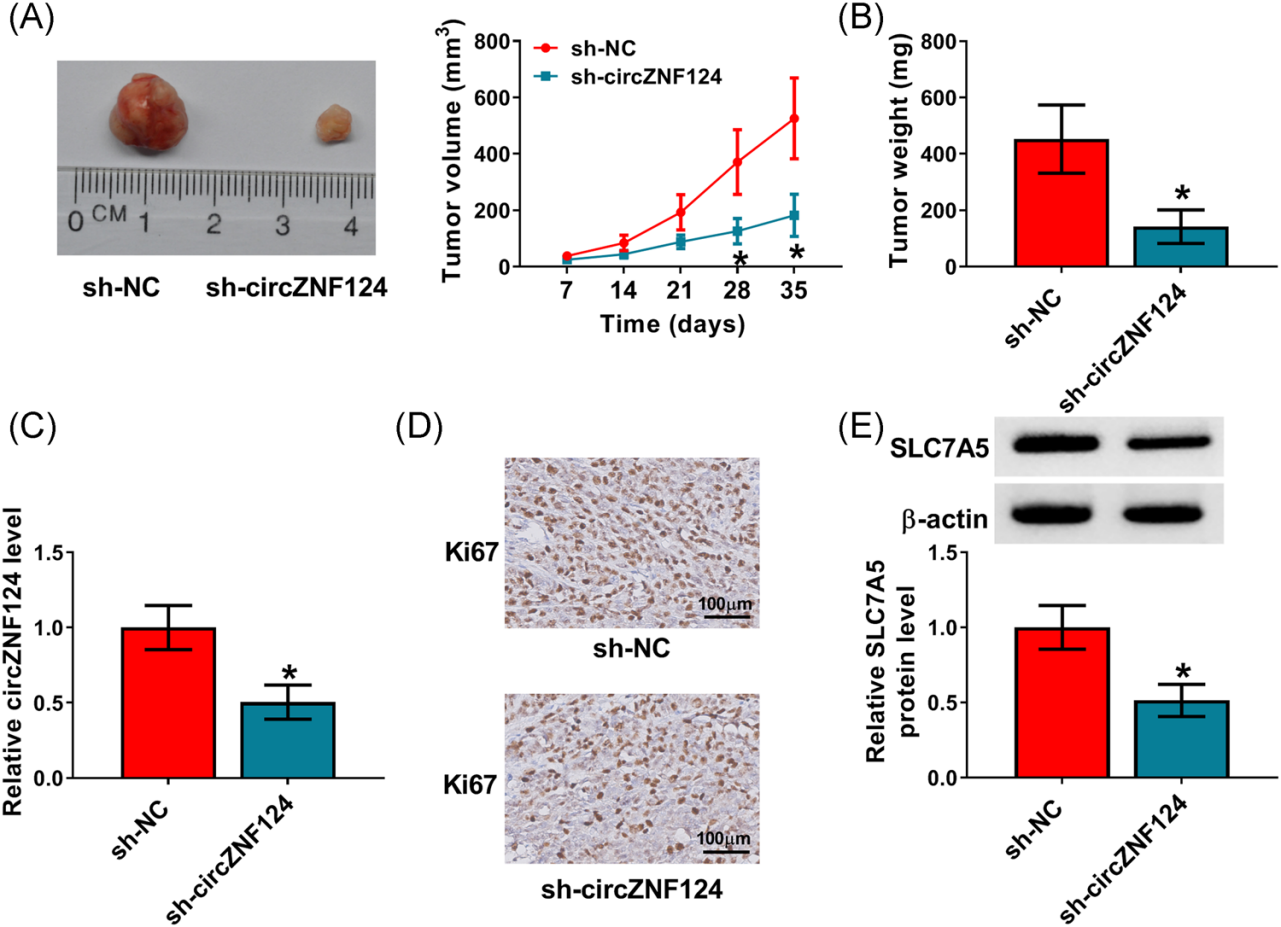
CircZNF124 downregulation repressed tumor growth by modulating miR‐199b‐5p and SLC7A5 in vivo. (A and B) The effect of circZNF124 silencing on tumor growth was determined by measuring the volume and weight of the forming tumors. (C) The impact of circZNF124 knockdown on the expression of circZNF124 was disclosed by qRT‐PCR in the forming tumors from sh‐circZNF124 and sh‐NC groups. (D and E) IHC assay and western blot analysis was used to reveal the effect of circZNF124 absence on Ki67 and SLC7A5 protein expression, respectively, in the forming tumors from sh‐circZNF124 and sh‐NC groups. The comparisons were assessed with two‐tailed Student's *t* tests in (A and B), and with Wilcoxon rank‐sum test in (C and E). *p < .05. CircZNF124, circRNA zinc finger protein 124; IHC, immunohistochemistry; qRT‐PCR, quantitative real‐time polymerase chain reaction; SLC7A5, solute carrier family 7 member 5

The effect of circZNF124 knockdown on tumor formation was further revealed in vivo. Data showed that circZNF124 silencing reduced tumor volume and weight (Figure 7A,B), suggesting circZNF124 knockdown repressed tumor growth. Additionally, we found that circZNF124 expression was weakly expressed in the neoplasms from sh‐circZNF124 group in comparison with its expression in the neoplasms from sh‐NC group (Figure 7C), showing that sh‐circZNF124 was effective in decreasing circZNF124 expression in the forming tumors. Furthermore, circZNF124 absence decreased the number of Ki67‐positive Ishikawa cells and downregulated SLC7A5 protein expression in the neoplasms from sh‐circZNF124 group when compared with its expression in those tissues from sh‐NC group (Figure 7D,E). These data explained circZNF124 knockdown repressed tumor growth by regulating miR‐199b‐5p and SLC7A5 in vivo.

## 4 DISCUSSION

EC chiefly occurs in postmenopausal women,^23^ and the prognosis of the advanced EC patients is still poor because of the immature treatment means. Hence, finding out the major regulators in EC genesis is necessary to identify novel therapeutic target for EC. CircRNA is more stable than its linear form due to the lack of 3′ poly (A) tail and 5′ cap,^24^ which means circRNA has the potential as a therapeutic target for cancers. In this study, we found circZNF124 regulated EC cell malignancy and leucine uptake by miR‐199b‐5p/SLC7A5 pathway.

Multiple evidence revealed that circRNAs were closely related to EC tumorigenesis. For example, circ_0000043 overexpression contributed to the proliferation and metastasis of EC cells.^25^ Circ_0109046 absence hindered cell growth and epithelial‐mesenchymal transition in EC.^26^ In another example, Li and his colleague^27^ indicated circ_0109046 depletion impeded cell aggressiveness and promoted cell apoptosis in EC in vitro. CircZNF124, a circRNA, has been reported to inhibit lung cancer processes by miR‐498/YES proto‐oncogene 1 axis.^28^ In the study of Kim et al.^29^ circZNF124 might be employed as a potential marker of Kawasaki disease as its significant dysregulation in the patients with Kawasaki disease. In this study, we found that circZNF124 regulated EC progression for the first time. Herein, we provided the evidence that circZNF124 was augmented in EC clinical samples and cells, and its expression was closely correlated with severity of EC as well as lymph node metastasis. CircZNF124 depletion repressed cell proliferation and aggressiveness, and hindered tumor formation. The energy synthesis before cell growth largely relies on exogenous amino acids, which are essential for protein synthesis and the activation of a variety of signaling pathways.^30^ Leucine, one of essential amino acids, was employed as a representative to be studied in EC development in this study. As a result, we found circZNF124 silencing restrained leucine uptake in EC cells. The results from our study suggested circZNF124 knockdown suppressed EC progression by impeding the uptake of cells to leucine.

CircRNA commonly acts as a competing endogenous RNA to interact with miRNA.^31^ Hence, to reveal the underlying mechanism through which circZNF124 mediated EC processes, the miRNAs interacted with circZNF124 were explored. Previous data showed miR‐199b‐5p contributed to tumor formation in cervical cancer by targeting kallikrein‐related peptidase 10.^32^ MiR‐199b‐5p repressed breast cancer tumorigenesis and angiogenesis via interacting with discoidin domain receptor tyrosine kinase 1 or activin receptor‐like kinase 1.^33^, ^34^ In renal cell carcinoma, downregulation of miR‐199b‐5p facilitated cellular proliferation and migration, and repressed cellular apoptosis.^35^ Based on the importance of miR‐199b‐5p in regulating cancer progression, we wondered whether it participated in circZNF124‐mediated EC development. We subsequently predicted miR‐199b‐5p expression through TCGA data set, and results showed miR‐199b‐5p expression was weakly downregulated (but not statistically significant) in UCEC tissues as compared with normal endometrial tissues. However, qRT‐PCR data showed the significantly low expression of miR‐199b‐5p in EC specimens in comparison with normal endometrial tissues. The above results suggested the prediction from TCGA data set did not always correspond to the actual situation. And miR‐199b‐5p was chosen for subsequent study. Herein, we found miR‐199b‐5p was negatively regulated by circZNF124, and decreased in EC cells. Moreover, miR‐199b‐5p expression was lower in stage III EC tissues than in stage I + II EC tissues. MiR‐199b‐5p mimics restrained cellular proliferation, metastasis, and leucine uptake in vitro, Furthermore, the repressive impact of miR‐199b‐5p inhibitors on circZNF124 absence‐mediated action implied that circZNF124 regulated EC progression by sponging miR‐199b‐5p.

Lots of cancer cells depend on system L transporter to obtain amino acids, and thereby the transporters are usually increased in cancer cells and play vital parts in cancer evolution.^36^ SLC7A5 is also named as L‐type amino acid transporter 1 and belongs to the system L transporter family that transports amino acids into cells, such as leucine, isoleucine, and valine.^36^ Coincidently, SLC7A5 was identified as a target mRNA of miR‐199b‐5p in this paper. In the report of Marshall et al.^37^  SLC7A5 was the most highly expressed in EC tissues among four LAT family members, and SLC7A5 depletion repressed cell growth and spheroid area in EC cells in vitro. Sato et al.^38^ also described SLC7A5 upregulation was positively associated with poor prognosis of EC patients. Additionally, SLC7A5 expression was higher in well‐differentiated EC than in poorly differentiated EC.^39^ In this study, the mechanism of SLC7A5 in regulating EC development was revealed for the first time. Herein, SLC7A5 expression was significantly increased in EC tissue samples and cells, but had little change in stage I + II EC tissues as compared with stage III EC tissues. Additionally, SLC7A5 promoted cell proliferation, metastasis and leucine uptake. SLC7A5 overexpression reversed circZNF124 silencing‐mediated EC progression, which meant that circZNF124 might promote EC progression by quickening the uptake EC cells to leucine through promoting SLC7A5 expression. We also discovered circZNF124 regulated SLC7A5 expression by interacting with miR‐199b‐5p in EC cells.

Taken together, circZNF124 was augmented in EC specimens and cells. CircZNF124 expression was related to severity of EC and lymph node metastasis. CircZNF124 depletion repressed EC cell malignancy and leucine uptake in vitro, and tumor formation in vivo. In mechanism, circZNF124 modulated SLC7A5 by binding to miR‐199b‐5p. Collectively, circZNF124 silencing inhibited EC tumorigenesis by hindering leucine uptake through miR‐199b‐5p/SLC7A5 axis. This study demonstrates that circZNF124 may be a diagnosis biomarker and circZNF124 inhibitors may be used to develop anticancer drug for EC.

## CONFLICT OF INTERESTS

The authors declare that there are no conflict of interests.

## AUTHOR CONTRIBUTIONS

Liuping Shu and Yan Peng designed the research study. Liuping Shu and Liyan Zhong performed the research and wrote the paper. Xi Feng and Lifu Qiao collected and analyzed the data. Lifu Qiao contributed essential reagents or tools. Xi Feng and Yi Yi edited the manuscript. All authors have read and approved the final manuscript.

## Supporting information

Supporting information.

Supporting information.

## Data Availability

The data sets used and analyzed during the current study are available from the corresponding author on reasonable request.
