# EXPRESSION OF CONCERN: CircZNF124 Regulates Cell Proliferation, Leucine Uptake, Migration and Invasion by miR‐199b‐5p/SLC7A5 Pathway in Endometrial Cancer

**DOI:** 10.1002/iid3.70222

**Published:** 2025-07-30

**EXPRESSION OF CONCERN**: L. Shu, Y. Peng, L. Zhong, X. Feng, L. Qiao and Y. Yi, “CircZNF124 Regulates Cell Proliferation, Leucine Uptake, Migration and Invasion by miR‐199b‐5p/SLC7A5 Pathway in Endometrial Cancer,” *Immunity, Inflammation and Disease* 9, no. 4 (2021): 1291‐1305, https://doi.org/10.1002/iid3.477.

This Expression of Concern is for the above article, published online on 19 June 2021 in Wiley Online Library (wileyonlinelibrary.com), and has been issued by agreement between the journal Editor‐in‐Chief, Marc Veldhoen; and John Wiley & Sons Ltd. The Expression of Concern has been agreed upon following the identification of duplicated images in Figures 4 G and 4H. The authors did not respond to requests for comment regarding the duplication. Although the conclusions are not believed to be affected, the journal has decided to issue an Expression of Concern to inform readers of the identified duplication.

