# Supplementary figures and images for: CircZNF124 regulates cell proliferation, leucine uptake, migration and invasion by miR‐199b‐5p/SLC7A5 pathway in endometrial cancer
Source: Immun Inflamm Dis. 2021 Jun 19;9(4):1291–305. doi: 10.1002/iid3.477 (PMC8589382; doi:10.1002/iid3.477)

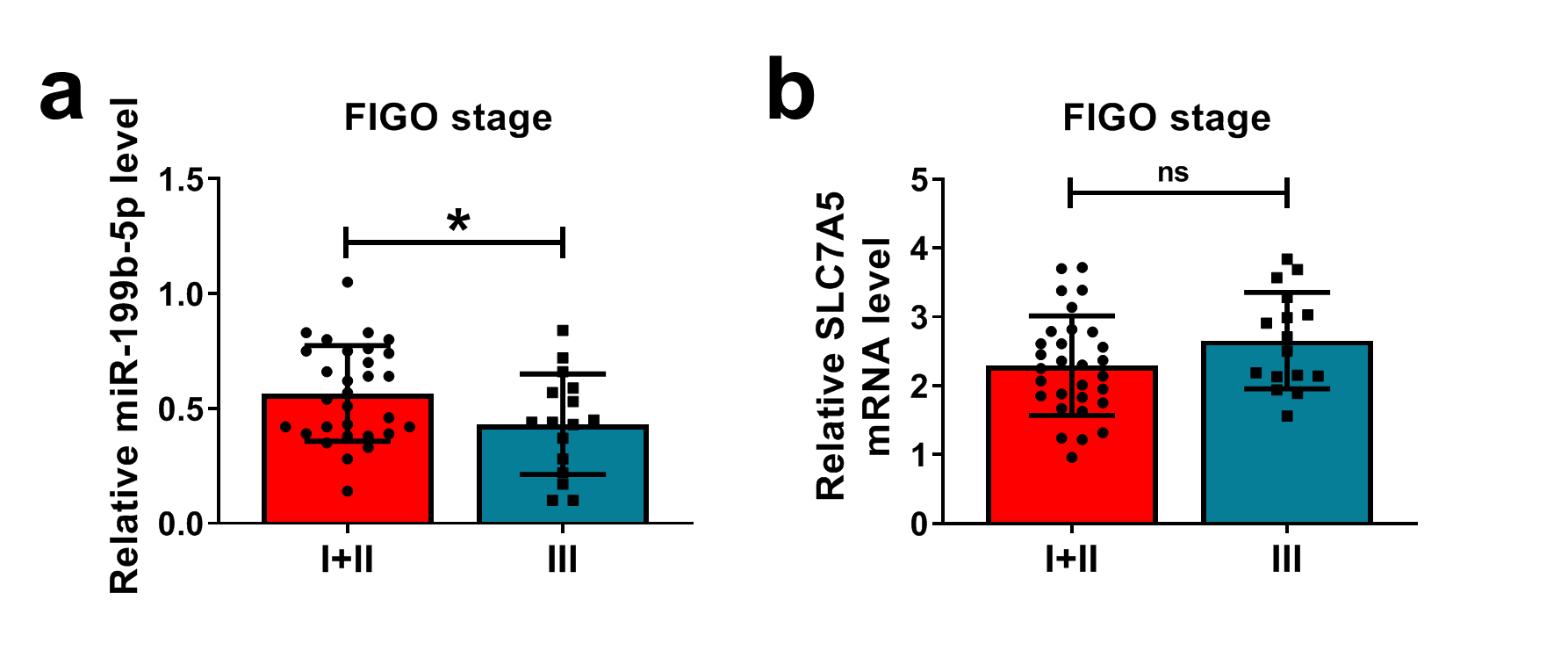

Supplement: Supplementary file 1 — Supporting information. [file IID3-9-1291-s002.tif]
