# Supplementary material for: CircZNF124 regulates cell proliferation, leucine uptake, migration and invasion by miR‐199b‐5p/SLC7A5 pathway in endometrial cancer
Source: Immun Inflamm Dis. 2021 Jun 19;9(4):1291–305. doi: 10.1002/iid3.477 (PMC8589382; doi:10.1002/iid3.477)
Supplement: Supplementary file 2 — Supporting information. [file IID3-9-1291-s001.docx]

**Table S1.** **The** **clinicopathologic features of endometrial cancer patients**

| Parameters | N=46 |
| --- | --- |
|  |  |
| Age,years |  |
| <60 | 26 |
| ≥60 | 30 |
| tumor size |  |
| <1 cm | 32 |
| ≥1 cm | 14 |
| FIGO stage |  |
| I+II | 30 |
| III | 16 |
| Lymph node metastasis |  |
| Negative | 34 |
| Positive | 12 |
